# Sex differences in immune cells: mechanistic perspectives and clinical correlates in physiology and disease

**DOI:** 10.3389/fimmu.2026.1650527

**Published:** 2026-02-17

**Authors:** Ximing Liao, Di Wu, Jing Gao, Fengyang Xie, Zhaoqi Li, Muyun Wang, Kun Wang, Yixuan Gao, Qiang Li, Wei Gao

**Affiliations:** 1 Department of Pulmonary and Critical Care Medicine, Shanghai East Hospital, School of Medicine, Tongji University, Shanghai, China; 2 Department of General Practice, Shanghai Jing’an District Caojiadu Community Health Center, Shanghai, China; 3 School of Medicine, Saint George’s University, Saint George's, Grenada; 4 Department of Gynecology, Shandong Provincial Hospital Affiliated to Shandong First Medical University, Shandong, China

**Keywords:** disease, immune cells, physiology, sex disparity, sexual hormone

## Abstract

For decades, sex disparities have been acknowledged to influence immune regulation in health and disease throughout the life span, and contribute to variations in epidemiology, pathophysiology, manifestation, progression, and therapeutic response in multiple disorders. However, the underlying cellular and molecular mechanisms governing these disparities remain understudied. This review articulates the effects of sex as critical regulators of the major causes of morbidity and mortality in diseases. We summarize the key factors driving sex differences, including sexual hormones and sex chromosomes, as well as elaborate how these factors influence physiology and disease, especially by modulating the function and fate of immune cells. Our aim is to disentangle the intricacies of sexually-differentiated immune responses within physiological and pathological contexts, thereby establishing the groundwork for precision medicine approaches customized to sex-specific requirements in clinical settings.

## 1 Introduction

Historically, the diagnosis, prevention, and treatment of disease has originated from research mostly conducted on male cells, male animals, as well as men (1). This is probably due to the safety consideration for women of childbearing age, and their offspring, which are always excluded from most clinical trials. Therefore, on the assumption that female and male cells and/or animals were biologically identical, medical research has concentrated on male physiology and evidence-based medicine depends on clinical studies done primarily in men. However, the neglect of sex disparities is sometimes harmful to human health. For example, in the United States, ten prescription drugs were withdrawn from usage between 1997 and 2001, among which eight had been proved to pose more risk for women than men (2). As a result, sex-based biology and medicine should be viewed as a core consideration in medical study and translational science, rather than a research area of interest.

Actually, accumulating evidence has revealed intricate differences in disease prevalence, severity, and outcomes between men and women (3). It has been noticed that males often present more intense inflammatory responses (4, 5), while females tend to exhibit a higher incidence of chronic autoimmune diseases (6, 7). Specifically, acute respiratory distress syndrome (ARDS) patients show male predominance (females accounting for 28%-38%) and consistently exhibit a higher annual mortality from ARDS compared to women (5, 8). Except for infants under 9 years old, women are less commonly affected by ARDS across all age groups, demonstrating a protective effect of sex disparity extending from puberty to post-menopause (5, 9). The recent coronavirus disease 2019 pandemic also underscore the sex difference in the outcomes, with men showing an elevated risk for severe infection and increased mortality rather than women (10). Conversely, over 50% of patients with autoimmune diseases (e.g., systemic lupus erythematosus [SLE] and Sjogren’s syndrome) are women, and these diseases are among the third most common disease categories (7). Furthermore, sex differences also exist in leukemia, particularly in acute myeloid leukemia, with females exhibiting lower incidence and mortality rates, along with lower healthcare expenditures (11, 12). Regrettably, most of the current understandings center on the epidemiological and clinical differences, with the underlying mechanisms remaining largely unexplored.

Efforts are being made to elucidate the factors contributing to sexual dimorphism in health and disease. Among them, sex hormones and chromosomal differences are thought to play significant roles. Study has pointed out that the zenith of rheumatoid arthritis onset in female aligns with menopause, characterized by a precipitous decline in estrogen level, implying an intricate link between sex hormones and disease etiology (13). Another representative example is bronchial asthma. Serum testosterone levels in both males and females are inversely correlated with the incidence (14, 15); while fluctuations in levels of estradiol and progesterone during the menstrual cycle are associated with the worsened symptoms in female patients (16–18). However, our understanding of key mechanisms shaping the sex dimorphism, especially in functional reprogramming of immune cells, remains surprisingly sparse (6, 19, 20). This review proposes a critical shift from descriptive cataloguing of immune sexual dimorphism toward a mechanistic, cell-type-resolved understanding. We uniquely systematically map sex differences across the immune continuum, linking hormonal and chromosomal drivers to functional outcomes; decipher the underlying molecular circuits (e.g., receptor signaling, transcription factors) that enact sex-specific functions in physiology and disease; and integrate the modulating effects of age, menopause, and environmental exposures. By synthesizing these layered insights, we provide a translational framework to rationally guide the development of sex-tailored immunotherapies and advance precision medicine.

## 2 Classification, distribution, function, and signaling pathways of sex-determining system

**Figure 1:**
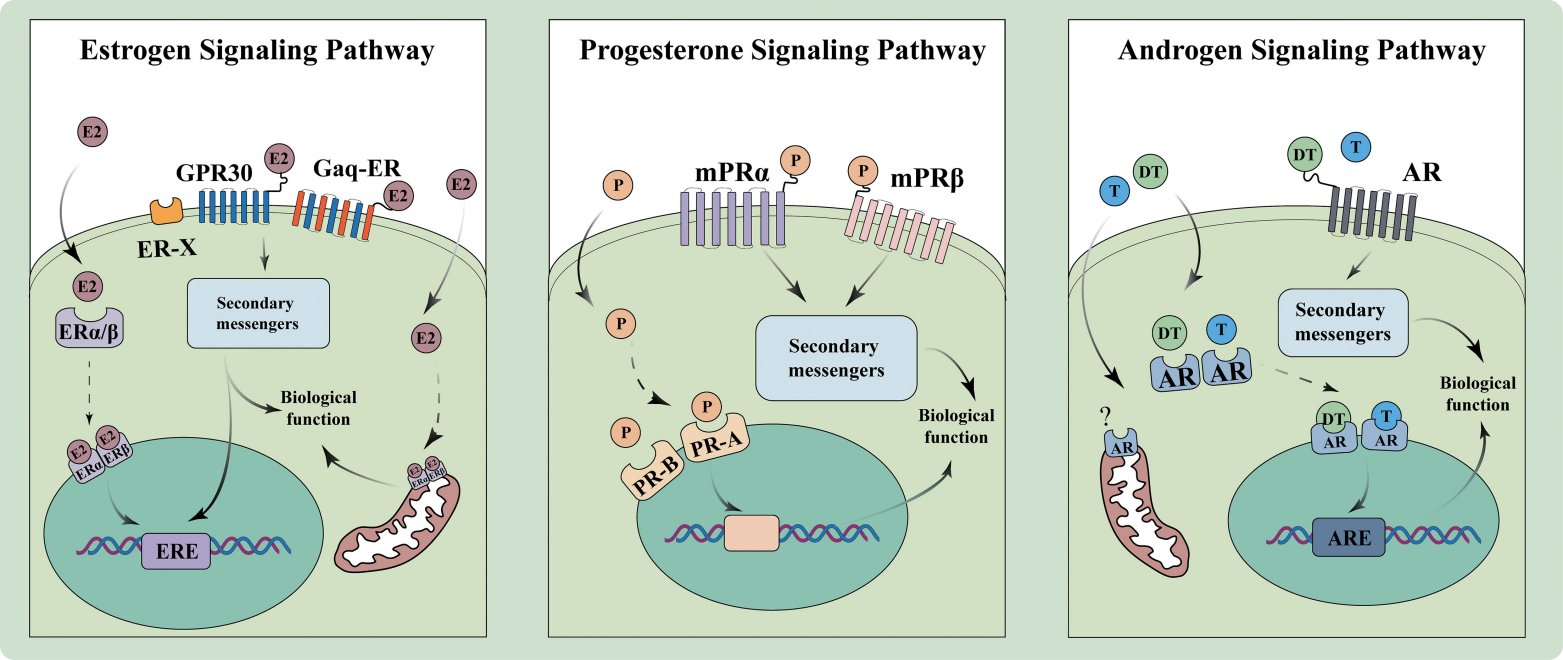
Schematic diagram of the sex hormone pathway. Signaling pathways of estrogen, progesterone, and androgen, including their interactions with receptors and subsequent biological functions. E2, Estradiol; ER, Estrogen receptor; GPR, G protein-coupled receptor; ERE, Estrogen response element; P, Progesterone; PR, progesterone receptor; mPR, membrane-progesterone receptor; T, Testosterone; DT, Dihydrotestosterone; AR, Androgen receptor; ARE, Androgen response element.

Sex-determining system linking to physiological differences is primarily divided into sexual hormones and sex chromosomes. The former mainly includes estrogen, progestogen, and androgen, all of which are steroid hormones synthesized from cholesterol. The initial step of the synthesis involves the conversion of cholesterol to pregnenolone by cytochrome P450 side-chain cleavage enzyme 11A1 on mitochondrial membrane. Pregnenolone subsequently follows two pathways: one is transformed into progesterone via 3β-hydroxysteroid dehydrogenase; the other is first converted to 17α-hydroxypregnenolone and then to dehydroepiandrosterone (DHEA) by cytochrome P450 17A1. DHEA can be further turned into androstenedione, which is then converted to testosterone and aromatized into estrogen by aromatase (21, 22). Meanwhile, sex chromosomes, X and Y, are essential in determining sex disparities: the Y chromosome, with *sex-determining Region Y* (*SRY*) gene, triggers male development and differentiation (23) (**Figure 1**).

### 2.1 Estrogen

Estrogen is a widely studied female hormone and exists in three forms: estrone, estradiol, and estriol. Among them, estradiol is the most potent and prevalent in premenopausal women, playing a crucial role in the development of reproductive organs and maintenance of tissue homeostasis. Besides, estradiol and estrone can be interconverted by 17β-hydroxysteroid dehydrogenase, and estriol is primarily produced from the other two during pregnancy (24). Estrogen predominantly derives from ovary, corpus luteum, and placenta during reproductive periods; while is generated from kidneys, adipose tissue, skin, and brain in pre-puberty or post-menopause stage. In females, estrogen level fluctuates significantly across different physiological periods, with estradiol peaking during the menstrual cycle, estriol rising during pregnancy, and overall estrogen level declining during menopause, transitioning from ovarian to peripheral tissue production (25). Interestingly, males maintain relatively consistent level of estrogen throughout their adult life, while post-menopausal women have less estrogen than men of the same age (26).

Estrogen exerts its effects via the classic nuclear receptors, estrogen receptor alpha (ERα) and estrogen receptor beta (ERβ), as well as novel membrane-associated receptors, G protein-coupled receptor 30, Gαq protein-coupled estrogen receptor, and ER-X. Notably, it has been reported that 88% of the human peripheral blood monocyte population is positive for ER (using a monoclonal antibody that recognizes both isoforms), providing direct evidence for ER expression in a key immune cell subset and supporting estrogen’s modulatory role in monocyte function. ERα is mainly expressed in uterus, liver, kidney, mammary gland, and adipose tissue, while ERβ can be found in lung, colon, cardiovascular system, and central nervous system (27, 28). For membrane-associated receptors, the former predominantly exists in adrenal medulla, renal pelvis, and ovary (28). Gαq-ER, though still in the early stage of research with its tissue distribution not yet fully established, is involved in rapid signal transduction via Gαq protein activation (29), while ER-X is known for its high-affinity binding in tissues like brain and uterus, particularly during postnatal development. Estrogen performs biological functions through different initiated pathways. Within the nuclear-initiated, estrogen interacts with ERα or ERβ, leading to translocation of the activated receptors from cell membrane to nucleus. The ERs then recruit transcriptional complexes and other co-regulatory factors to specific DNA sequences known as estrogen responsive elements, thereby modulating target gene transcription (30). This genomic action often involves the formation of ER homodimers or heterodimers on DNA. In addition, ERs also orchestrate a cascade of rapid estrogen responses through membrane-initiated signaling, far outpacing the above transcriptional process (31). These non-genomic signals are mediated by membrane-associated receptors and can rapidly activate pathways such as extracellular signal-regulated kinase)/mitogen-activated protein kinase, protein kinase C (elevating intracellular Ca^2+^), JNK/phosphatidylinositol 3 kinase, and cAMP-dependent transcription, which regulate critical cellular processes including apoptosis. At the receptor level, ERα and ERβ exhibit similar affinities for estradiol, whereas ERβ can antagonize and negatively regulate the function of ERα (32). ERα and ERβ are able to form homodimers or heterodimers within the cell nucleus; besides, ERα can also form heterodimers with androgen receptor (AR), thereby modulating the transcriptional activity (33).

Notably, estrogen modulates the functions of specific immune cell subsets (neutrophils, macrophages, CD4^+^ T cells) through these ER-mediated signaling pathways by regulating key transcription factors including *peroxisome proliferator-activated receptor gamma* (*PPAR-γ*); shaping cell-type dependent cytokine profiles such as promoting the secretion of interleukin (IL) -4, IL-6, and interferon gamma (IFN-γ) while inhibiting anti-inflammatory IL-10 and regulating IL-17A; and modulating the expression of surface receptors including C-X-C chemokine receptor type 2 (CXCR2), integrin alpha M, and toll-like receptor (TLR) 4. Detail mechanisms of ER-mediated sex-specific regulation in immune cells are elaborated subsequently.

### 2.2 Progesterone

Progesterone, also the principal female hormone, is integral to various physiological processes, including embryogenesis, puberty, menstrual cycle, and pregnancy. While primarily synthesized in the ovaries, a small amount of progesterone is also produced by the brain and adipose tissue. During reproductive years, progesterone fluctuates significantly, peaking in the luteal phase of menstrual cycle and rising substantially during pregnancy when the placenta takes over its production from the corpus luteum. After menopause, progesterone level drops sharply, with limited production continuing in peripheral tissues like adrenal glands. Beyond its reproductive role, progesterone is critical for maintaining tissue homeostasis and modulating the immune system, particularly preventing fetal rejection during pregnancy (34).

Functionally, progesterone exerts its effects by binding to specific receptors: progesterone receptor (PR) and, in some contexts, glucocorticoid receptor (GR). The PR is encoded by progesterone receptor gene, located on chromosome 11q22. PR-A and PR-B are two main isoforms of nuclear PR, which differ in structure and function. PR-B generally acts as a positive regulator of progesterone, while PR-A can modulate and, in some cases, antagonize the effects of PR-B (35). These receptors, upon binding to progesterone, translocate to the nucleus and interact with specific progesterone response elements on DNA, influencing the transcription of genes related to cell proliferation, differentiation, and maintenance of the reproductive tissues (34). In addition, progesterone also signals through membrane PRs (mPRs), such as mPRα and mPRβ, which mediate rapid, non-genomic actions. The receptors activate G-proteins, initiate intracellular signaling cascades, and affect key cellular processes like smooth muscle contraction, immune regulation, and apoptosis (36). For immune regulation, progesterone acts on macrophages and regulatory T cells (Tregs) via PR-mediated pathways, which involves inhibiting *nuclear factor kappa-B* (*NF-κB*) and upregulating *forkhead box P3* (*FOXP3*) at the transcription factor level; reducing the release of pro-inflammatory cytokines including IL-6 and tumor necrosis factor-α while enhancing anti-inflammatory IL-10; and suppressing the formation of neutrophil extracellular traps (NETs) by neutrophils. Sex-biased immunomodulation of progesterone on cells is later discussed.

### 2.3 Androgen

Androgens are a group of hydrophobic steroid hormones including testosterone, androstenedione, dihydrotestosterone and DHEA, and play an important role in maintaining male characteristics and physiological functions (37). Of these, testosterone is the most important in men, since it ensures the ability to produce sperm and participation in sexual activity. In addition, testosterone can also affect men’s physical strength, brain function, and heart health, which is equally important for women and helps maintain their overall health and sexual desire. In mammals, androgens are mainly synthesized and secreted by interstitial cells of the testis, with small amounts also produced by adrenal cortex and ovary. The level of androgen reaches peak during adolescence, and gradually decreases with age, especially over the age of 50, when its production declines by about one-third (37).

AR is a member of the ligand-activated transcription factor superfamily and mediates the effects of androgen. Among the hormones, only testosterone and dihydrotestosterone can bind to AR with high specificity and affinity, with the latter having approximately four times higher affinity than the former (38). Androgen exerts functions through both genomic and non-genomic pathways. Within the former, conformational change in the AR induced by ligand binding leads to the translocation of the receptor from cytoplasm to nucleus. The AR then acts as a homodimer and directly interacts with DNA at the androgen response element in the target gene regulatory region. In contrast, the non-genomic response occurs efficiently, and independently of gene transcription or protein synthesis, involving interactions with cell membrane-associated signaling molecules such as membrane receptors, ion channels or cytoplasmic regulatory proteins (38). Specifically, testosterone (the major androgen) mediates sex-biased immune cell functions in natural killer (NK) cells, CD8^+^ T cells, and neutrophils via AR-dependent genomic and non-genomic pathways, which involves suppressing *interferon regulatory factor 8*, activating *forkhead box O1*, and modulating *T-Box transcription factor 21* at the transcription factor level; inhibiting the secretion of pro-inflammatory cytokines such as IL-1β and type I interferon (IFN)-γ; and regulating surface receptor expression by upregulating programmed death-ligand 1 (PD-L1) and downregulating *natural killer group 2D*. AR-dependent sex-specific functions in NK, CD8^+^ T cells, and neutrophils are systematically detailed thereafter.

It is noteworthy that, multiple sex hormone receptors (ERα/β, PR-A/B, and AR) all belong to the nuclear receptor superfamily and share common structural domains: the C-terminal ligand-binding domain, the variable N-terminal with a transcriptional activation function domain, the central DNA-binding domain, and the hinge region. The second transcriptional activation function domain is located within the C-terminal ligand-binding domain region and acts as an additional transcriptional activation area upon ligand binding (39). These structural similarities enable interactions between various sex hormones at multiple levels.

### 2.4 Sex chromosome

Sex chromosomes, comprising X and Y chromosomes, genetically determine the sex disparities from reproductive organs to the appearances/behaviors of adult individuals. The differentiation between X and Y chromosomes arises from their distinct evolutionary paths. X chromosome is larger and contains more amounts of genes, many of which are essential for basic cellular functions and immune responses (40). In contrast, the Y chromosome is smaller, expressing fewer genes, which are primarily related to male sex determination and spermatogenesis. Sex chromosomes can be classified into gonadal and non-gonadal actions. Due to the previous discussion on the effects of sex hormones, we mainly focus on the non-gonadal actions of sex chromosomes here.

#### 2.4.1 X chromosome

X chromosome plays a critical role in numerous physiological and immunological processes except for the influence on sex determination. In somatic cells of XX individuals, one X chromosome undergoes transcriptional silencing, ensuring that most X-linked genes are expressed equally from the single active X chromosome in both XX and XY cells. However, several genes (up to 20%) escape the inactivation, resulting in their more expression in XX cells compared to XY cells. Notable examples include *TLR*, histone demethylases *lysine demethylase 5C and 6A*, the translation initiation factor *eukaryotic translation initiation factor 2 subunit 3 X-linked*, and RNA helicase *DEAD-box helicase 3 X-linked*. Specifically, *Tlr7* located on X chromosome is crucial for recognizing viral RNA and initiating antiviral response. Overexpression of TLR7 and TLR8 in females can lead to stronger immune responses and a higher prevalence of autoimmune diseases (40), like systemic lupus erythematosus (41). Another case is that patients with systemic sclerosis exhibit a higher ratio of X chromosome inactivation and decreased regulatory T cell activity (42). Meanwhile, significant difference in susceptibility to various infectious and inflammatory diseases has been revealed between prepubertal boys and girls, underscoring the critical role of X chromosome (43, 44).

#### 2.4.2 Y chromosome

Y chromosome is essential for male sex differentiation and also participates in various physiological processes, though its function is more restricted compared to X chromosome due to lower gene content. The most important action of Y chromosome is driven by *SRY* gene, triggering the development of testes in males. Interestingly, *SRY* can also be expressed in brain, particularly in catecholaminergic cells and neurons, suggesting a modulatory role in physiological conditions, such as hypertension (45). Notably, the limited number of genes on Y chromosome has made it challenging to investigate its non-gonadal effects. Study on XY mice with distinct Y chromosome variants have demonstrated significant variations in the severity of autoimmune diseases, highlighting the function of Y chromosome in immune responses (46). Y chromosome has also been implicated in modulating CD4^+^ T cells, further illustrating its contribution to the sex dimorphism in immunology (47).

Collectively, sex hormones act via specific receptors and signaling axes, while sex chromosomes provide genetic underpinnings for sex disparities—these factors together shape immune cell sexual dimorphism, as detailed in Section 3.

## 3 Sexual dimorphism of different cell types in physiological and pathological conditions

The immune system serves as a critical regulator of host physiology, necessitating specialized control across different tissues, ages, and sexes. This tailored regulation is believed to explain the variations in disease incidence, tropism and severity between male and female. This section examines the sex-specific biological factors that may contribute to differences in physiological and pathological conditions. It is noteworthy that, while environmental and social factors, such as occupational hazards, habits and social stresses, can influence these discrepancies, our focus is mainly on studies at cellular and *in vivo* levels.

### 3.1 Neutrophil

**Figure 2:**
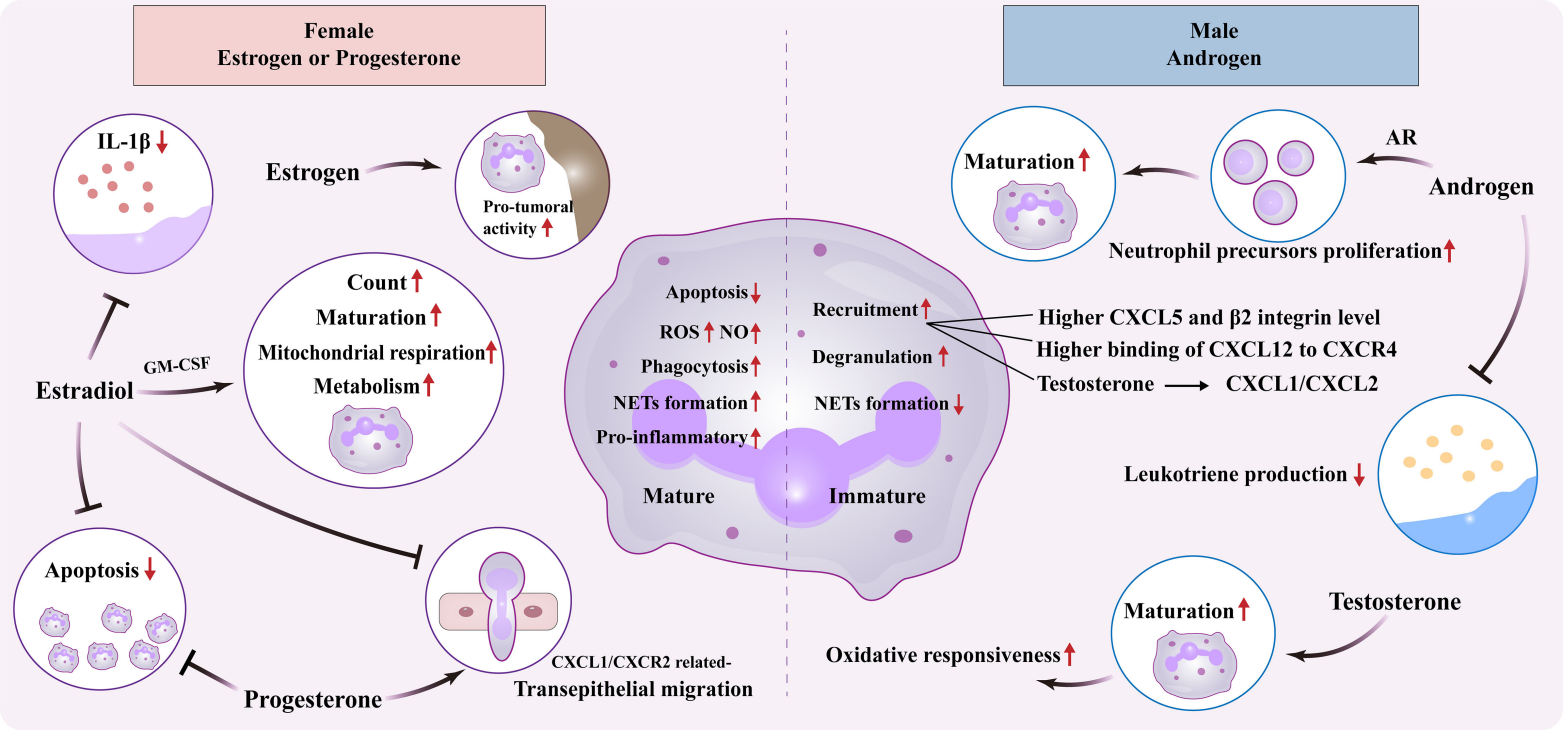
Sexual dimorphism of neutrophil. Effect of female estrogens or progesterones and male androgens on neutrophil biology, including maturation, apoptosis, metabolism, and inflammatory/tumor activity. ER, Estrogen receptor; AR, Androgen receptor; GM-CSF, Granulocyte-macrophage colony-stimulating factor; ROS, Reactive oxygen species; NO, Nitric oxide; IL, Interleukin; CXCL, Chemokine C-X-C motif ligand; CXCR, C-X-C chemokine receptor; NETs, Neutrophil Extracellular Traps.

Polymorphonuclear leukocytes (neutrophils) are the predominant type of leukocytes in peripheral blood and known to play a key role in the defense of pathogen invasion and external stimuli. As frontline immune responder, neutrophils are rapidly recruited to the sites of infection, and combat invading pathogens via multiple mechanisms, such as phagocytosis, degranulation, secretion of reactive oxygen species (ROS), and formation of NETs. However, excessive accumulation and dysfunction of these cells lead to abnormal inflammatory process and may ultimately determine patient outcomes (48). Meanwhile, neutrophils expresses both ER and AR, which largely affect its quantity, function, and fate (49). This aspect warrants further investigation for deeper insight in sex-specific differentiation of neutrophils (**Figure 2**) (**Table 1**).

**Table 1:** Sex difference of neutrophil.

Etiology	Model/Source	Effects	References
Physiology	Human blood neutrophil	Immature profile with diminished NETs formation in male	Blazkova et al., 2017 (50)
Physiology	Human blood neutrophil	Mature profile with activated IFN pathway and proinflammatory response in female, while increased mitochondrial mass and respiration in male	Gupta et al., 2020 (51)
Systemic juvenile idiopathic arthritis	Human blood neutrophil	Immature signature in female with suppressed therapeutic responsiveness of anti-IL-1R	Prada-Medina et al., 2020 (52)
Influenza vaccine	Human blood neutrophil	E2 triggers GM-CSF production and neutrophil maturation	Furman et al., 2014 (54)
Physiology	*AR*^-/-^ mouse	Androgen-AR promotes precursor proliferation and neutrophil maturation	Chuang et al., 2009 (55)
Peritonitis and pleurisy	Rat, T cell-deficient mouse	Increased neutrophil recruitment and exacerbated inflammatory damage in male	Scotland et al., 2011 (57)
Ischemia/reperfusion	Rat, mouse, human skin blisters	Increased neutrophil recruitment in male	Madalli et al., 2015 (58)
Inhalation lung injury	Mouse	Increased recruitment and activation of neutrophil in male	Chatterjee et al., 2023 (59)
Post-weaning	Mouse	Estrogen/ERα/CXCR2 signaling governs neutrophil activity, thus fostering inflammation and adipocyte repopulation	Lim et al., 2020 (60)
*C. albicans* vaginal infection	*Cxcr2*^−/−^ mouse	E2 disrupts while progesterone promotes neutrophil transepithelial migration	Lasarte et al., 2016 (61)
Bacterial prostatitis	Mouse	Testosterone increases CXCL1/CXCL2 expression and neutrophil recruitment with an N2-like phenotype	Scalerandi et al., 2018 (62)
*E. coli* endotoxin	Rat	Higher capacity for endotoxin phagocytosis in reproductive-age female	Spitzer & Zhang, 1996 (63)
*Staphylococcus aureus* infection	Mouse	Enhanced ROS production and bactericidal capacity in female	Pokhrel et al., 2020 (64)
Myocardial infarction	Mouse	Tend to generate nitric oxide, ROS and phagocytosis to remove necrotic tissue in female	DeLeon-Pennell et al., 2018 (65)
Sepsis	Mouse	Females exhibit a propensity for NETosis, whereas males display a predilection for elastase degranulation	Lu et al., 2021 (66)
Acute colitis	Rabbit	Elevated pro-inflammatory prostaglandin in male	LeDuc & Nast, 1990 (67)
Physiology	Human blood neutrophil	Androgen inhibits leukotriene production in male	Pergola et al., 2011 (68)
Spontaneous lupus	Mouse	Estrogen increases neutrophil count and activity	Dai et al., 2017 (69)
Aneurysm rupture	Mouse	Estrogen promotes NETosis and subsequent aneurysm rupture	Patel et al., 2023 (70)
Pregnancy	Human blood neutrophil	Progesterone suppresses NETs formation	Giaglis et al., 2016 (71)
Pregnancy	Human blood neutrophil	Delayed apoptosis with physiological neutrophilia in pregnancy female	Tulchinsky & Hobel, 1973 (75); Watson et al., 1999 (76)
Physiology	Human blood neutrophil	Decreased spontaneous apoptosis in female of reproductive age; estradiol and progesterone decrease neutrophils apoptosis	Molloy et al., 2003 (77)
Psoriatic inflammation	Mouse	E2 inhibits IL-1β generation and IL-17A-mediated psoriatic inflammation	Adachi et al., 2022 (72)
Mammary tumor	Mouse	Estrogen promotes tumor growth by upregulating the pro-tumoral activity of neutrophil	Lim & Lin, 2021 (78)
Mammary tumor	Mouse	Estrogen fosters pro-tumoral microenvironment	Chung et al., 2017 (73)
Melanoma tumor	Mouse	Impaired maturation and oxidative responsiveness of neutrophil in both female and castrated male	Markman et al., 2020 (74)

NETs, Neutrophil Extracellular Traps; IFN, Interferon; IL, Interleukin; GM-CSF, Granulocyte-Macrophage Colony-Stimulating Factor; ER, Estrogen Receptor; AR, Androgen Receptor; CXCL, C-X-C Motif Chemokine Ligand; Cxcr2:C-X-C Chemokine Receptor 2; ROS, Reactive Oxygen Species; E2, Estradiol.

#### 3.1.1 Neutrophil maturation

Neutrophil maturation, encompassing a multi-stage process from myelocyte to segmented neutrophil, is critical for immune defense. There are opposite opinions about its difference between men and women. On study reported an increased immature-like forms of neutrophil in healthy adult males compared to females of the same age, as manifested by the diminished capacity in NETs formation upon distinct stimuli (50). Another research also demonstrated that healthy young females possessed a mature neutrophil profile characterized by activated IFN pathway and proinflammatory responses, while males exhibited an immature-like genotype in circulating neutrophils (51). However, in patients with systemic juvenile idiopathic arthritis, peripheral neutrophils from females exhibit controversially higher immature signatures, which affects the therapeutic responsiveness of anti- IL-1R (52).

The underlying mechanism may be related to a key modulator of neutrophil maturation, granulocyte-macrophage colony-stimulating factor (GM-CSF), which was observed to be higher in young adult females than males, followed by diminishing after menopause (53). In addition, estradiol is capable of triggering GM-CSF production, thus influencing neutrophil maturation (54). Notably, neutrophil maturation shows conflicting sex biases: healthy adult males exhibit more immature phenotypes, while females with systemic juvenile idiopathic arthritis present higher immature signatures, likely due to disease-induced inflammatory cues overriding basal sex hormone regulation. Yet, this opinion requires further validation. Meanwhile, androgens also wield significance in shaping neutrophil maturation. Mice lacking ARs unveiled neutropenia owing to reduced proliferation of the precursor cells, ultimately compromising the host defense against microbial infections (55). While recent evidence pointed that, during urinary tract infection, androgen exposure raises the proportion of immature neutrophils in the kidney to ~50-60% (vs. <25% in controls). Selective deletion of ARs in myeloid cells largely rescues this defect, reducing immature neutrophils to ~25-30% (56).

#### 3.1.2 Neutrophil recruitment

Prior studies have identified certain patterns of sex differences in neutrophil recruitment or migration. In comparison with females, male rodents exhibited increased neutrophil recruitment and exacerbated inflammatory damage upon acute exposure to bacteria or other irritants (57). This sex-based disparity in neutrophil kinetics could be explained by both more production of chemokine C-X-C motif ligand 5 (CXCL5) in the circulation, tissue, bone marrow and higher expression of β2 integrin on neutrophils of males but not females in the animal model of ischemia/reperfusion (58). In a model of inhalation lung injury, Cl_2_ gas exposure also induced higher susceptibility and mortality in male mice than females, along with increased recruitment and activation of neutrophils. The differential binding of CXCL12 to its receptor, CXCR4, might be responsible for the sexual dimorphism in neutrophil migration and immune response (59).

When it comes to the specific mechanism, sex hormones exert distinct regulatory effects on neutrophil recruitment. In the study of mammary involution post-weaning, estrogen was uncovered to govern neutrophil activity in mammary tissue via ERα-mediated CXCR2 signaling, thus fostering inflammation and adipocyte repopulation (60). In addition, estradiol could disrupt while progesterone promoted the CXCL1/CXCR2 axis, followed by coordinating neutrophil transepithelial migration into the vagina (61). As for male hormones, testosterone was able to increase the recruitment of neutrophils to infection sites by upregulating expression of CXCL1/CXCL2. However, these neutrophils display an N2-like phenotype characterized by reduced efficiency in bacterial killing and heightened expression of immunomodulatory molecules like IL-10 and transforming growth factor-1 (62).

#### 3.1.3 Neutrophil function

Sex-dependent performance of neutrophil function has long been a subject of interest and is likely closely associated with certain diseases. During the battle to pathogens, neutrophils from reproductive-age female rats exhibited a greater capacity for endotoxin phagocytosis compared to age-matched male rats of the same strain, as well as those before or after reproductive age (63). Female mice manifested elevated serum levels of complement C3 and higher expression of complement receptor 3 on the neutrophils, thus enhancing their ROS production and killing capacity upon *Staphylococcus aureus* challenge (64). Another study indicated that female neutrophils effectively removed necrotic tissue following myocardial infarction via generating nitric oxide and ROS, as well as phagocytosis, protecting from collateral damage incurred by degranulation of proteolytic enzymes (65). Additionally, female mice tended to use NETosis, which forms a net outside the cell to capture pathogens (66). Comparatively, male neutrophils have their own characters. In male post-myocardial infarction, neutrophils eliminated necrotic tissue mainly through CD36-dependent degranulation of matrix metalloproteinase-9, leading to more excessive damage to the myocardium compared to the females (65). Similarly, during the antiviral immunity, neutrophils of male mice also exerted a killing effect through degranulation of elastase, but could be more prone to aging (66). As important pro-inflammatory mediators derived from arachidonic acid, prostaglandin is demonstrated to be elevated in males, while leukotriene is increased in females (67). Wherein, male neutrophils display a stronger ability to generate prostaglandin, likely due to heightened cyclooxygenase-2 expression linked to increased nuclear factor kappa-B activation during acute inflammation compared with the females (67). Moreover, androgens have been observed to inhibit leukotriene production in human neutrophils, resulting in its lower level in males (68). Collectively, these findings on neutrophil function may explain the differences in manifestations and clinical outcomes between men and women in certain diseases, like infection.

The regulation of neutrophil function by estrogen and its impact on diseases have been subject to ongoing debate. Male neutrophils exhibited increased mitochondrial mass and respiration, akin to earlier forms of neutrophils/myeloid cells. However, when treated with estradiol, these traits were reversed, suggesting the discrepancy in neutrophil bioenergetics between male and female (51). Meanwhile, estrogen administration dramatically increases the count and activity of neutrophils in the spleen of C57BL/6 mice, which might link to the potential promotion of spontaneous lupus (69). Intracranial aneurysm is more prevalent in women than men, while estrogen can protect against its progression to rupture via NETs formation (70), while progesterone has been shown to have a suppressive effect on NETs formation (71). Besides, estradiol could inhibit IL-1β generation from neutrophils and macrophages, which subsequently suppressed IL-17A-mediated psoriatic inflammation in the mouse model (72). The modulatory action of estrogen on neutrophil function appears to involve in tumor development. During the mammary involution, estrogen was reported to increase the expression of pro-tumoral cytokines/chemokines, and tissue-remodeling enzymes in the neutrophils, which created a pro-tumoral microenvironment (73). In addition, almost two-thirds of melanoma deaths are men. Researcher have discovered impaired maturation and oxidative responsiveness of neutrophils in both female and castrated male mice. This was correlated with increased melanoma tumor burden within the lungs and could be reversed by administering a physiological level of testosterone (74).

#### 3.1.4 Neutrophil fate

It has been noticed by previous studies that neutrophils in women at full-term pregnancy demonstrate a remarkable delay in apoptosis along with physiological neutrophilia (75, 76). Thereby raises the interest of Molloy’s team in investigating the effect of female hormones on the fate and function of neutrophils (77). They validate a decreased spontaneous apoptosis in women of reproductive age compared to men, which can be further delayed by estradiol and progesterone at physiologic doses. The regulatory action of female sex steroids relies on reduced release of cytochrome c from the mitochondria and subsequent changes in caspase cleavage and activity. Conversely, prolonged estrogen treatment in tumor-bearing mice significantly hampers the output from bone marrow, shortens the life span, and upregulates the pro-tumoral activity of neutrophils, thus promoting the growth of mammary tumor (78).

Collectively, estrogen regulates neutrophil recruitment and maturation via ERα/CXCR2 and GM-CSF-dependent pathways, while androgens impair neutrophil functional maturation through AR signaling; progesterone suppresses NETs formation, together shaping the sex-specific functional bias of neutrophils. These sex-dependent differences (e.g., enhanced phagocytosis and NETosis in females, immature phenotype and elastase degranulation in males) underlie the higher prevalence of autoimmune diseases in females and increased susceptibility to severe infections (e.g., ARDS, novel coronavirus pneumonia) and melanoma in males.

### 3.2 Eosinophil and basophil

Eosinophils orchestrate allergic response, anti-parasitic defense, and inflammatory modulation, whereas basophils initiate allergic cascades through histamine and inflammatory mediators release. Despite their immunological importance, research on sex differences in these granulocytes remains limited, with mechanistic insights particularly lacking (**Table 2**).

**Table 2:** Sex difference of NK, macrophage, eosinophil and basophil.

Cell	Etiology	Model/Source	Effects	References
NK	Myocarditis	Mouse	Estrogen inhibits cardiac IFN-γ^+^ NK cell infiltration in female	Zhou et al., 2018 (124)
	Parkinson	Mouse	Heightened NK cell receptor expression and IFN-γ production in young female	Menees et al., 2021 (125)
	Prostate cancer	Human PCa cell line	Dihydrotestosterone reduces the cytotoxicity of NK cells	Tang et al., 2022 (126)
	Bladder cancer	Human BCa cell lines, human NK cell line	Anti-androgen therapy or AR depletion enhances the tumor-killing efficacy of NK cells	Liu et al., 2022 (127)
Macrophage	Q fever	Human peripheral macrophage	Estrogen enhances inflammatory profile of macrophages while testosterone fosters the anti-inflammatory response	Gay et al., 2021 (129)
	Peritonitis and pleurisy	Rat, mouse	E2 or progesterone enhances the phagocytic activity of peritoneal macrophage	Chao et al., 1996 (130); Scotland et al., 2011 (57)
	Atherosclerosis	Human, *Ldlr*^-/-^ mouse	Estrogen-ERα inhibits TLR4 signaling activation and macrophage-associated inflammation	Meng et al., 2023 (131)
	Pregnancy loss	Human, mouse	E2 negatively modulates TLR4-induced M1 polarization and increases Th2 immune response	Lou et al., 2023 (132)
	*Lipopolysaccharide* infection	Human peripheral macrophage	Allopregnanolone inhibits TLR4/TLR7 activation and chemokine/cytokine generation in female	Balan et al., 2022 (133)
	Non-alcoholic steatohepatitis	Mouse	Estrogen-ERα decreases the recruitment and activation of M1 macrophage	Shu et al., 2022 (134)
	Postmenopausal chronic stress	Rat, mouse	E2 promotes macrophage polarization from M1 to M2 phenotype	Hou et al., 2021 (135)
	Asthma	Mouse	Estrogen-ERα enhances IL-4-induced M2 polarization	Bang et al., 2011 (137); Keselman et al., 2017 (136)
	Asthma	Human peripheral macrophage	Stronger IL-4 responsiveness with increased monocyte recruitment and M2 polarization in female	Becerra-Díaz et al., 2021 (138)
	*Streptococcus agalactiae* pneumonia	Mouse	Higher level of chromosome X-linked miR-223-3p which negatively correlates with M1 phenotype in female	Deny et al., 2022 (141)
Eosinophil	Asthma	Mouse	Estrogen-ERα amplifies Th2 cell polarization and exacerbates eosinophilic inflammation	Lauzon-Joset et al.,2020 (81); Cephus et al., 2021(80)
	Breast tumor	Mouse, mouse breast cancer line	Estrogen-ERα suppresses tumoral and circulating eosinophils, promoting tumor growth	Artham et al., 2024(82)
Basophil	Idiopathic anaphylaxis	Human	Null effect of estrogen/progesterone on basophil histamine release	Slater et al., 1987(83)
	Allergic disease	Human	Androgen modulates basophil functionality and suppresses Th2 cytokine production	Grobe et al., 2020(84)

NK, Natural Killer cell; IFN, Interferon; PCa, Prostate Cancer; Bca, Breast Cancer; AR, Androgen Receptor; ER, Estrogen Receptor; E2, Estradiol; TLR, Toll - like receptor; IL, Interleukin; Th2, T helper 2.

Pioneering observation in 1955 reveals sexually dimorphic eosinophil fluctuations in guinea pigs during physiological cycles, suggesting sexual hormonal modulation of the generation, maturation, and survival of eosinophils (79). Subsequent rodent studies identify estrogen’s regulatory capacity: it amplifies T helper 2 (Th2) cell polarization in allergic airways by enhancing IL-33 release from epithelial cells, thereby exacerbating eosinophilic inflammation via group 2 innate lymphoid cell activation (80, 81). Paradoxically, estrogen exhibits tumor-promoting effects in breast cancer models by suppressing tumoral and circulating eosinophils, which can be reversed with ERα blockade (82). This aforementioned differences in estrogen’s effects on eosinophils may stem from tumor microenvironment-specific signaling pathways or cell subset heterogeneity.

Basophil regulation displays contrasting hormonal dynamics. Clinical studies in idiopathic anaphylaxis patients demonstrate null effects of estrogen/progesterone on basophil histamine release (83). Conversely, androgens modulate basophil functionality indirectly via peripheral blood mononuclear cell-derived soluble factors, suppressing Th2 cytokine production and potentially explaining male-female disparities in allergic disease prevalence (84). In short, estrogen modulates eosinophil function via ERα-mediated IL-33/Th2 axis activation, while androgens indirectly regulate basophil functionality through suppressing Th2 cytokine production; progesterone shows no significant effect on basophil histamine release. This hormonal regulation explains the female predominance in allergic asthma (linked to eosinophilic inflammation) and the tumor-promoting effect of estrogen in breast cancer (via eosinophil suppression), as well as sex differences in the prevalence of allergic diseases.

### 3.3 T cell

**Figure 3:**
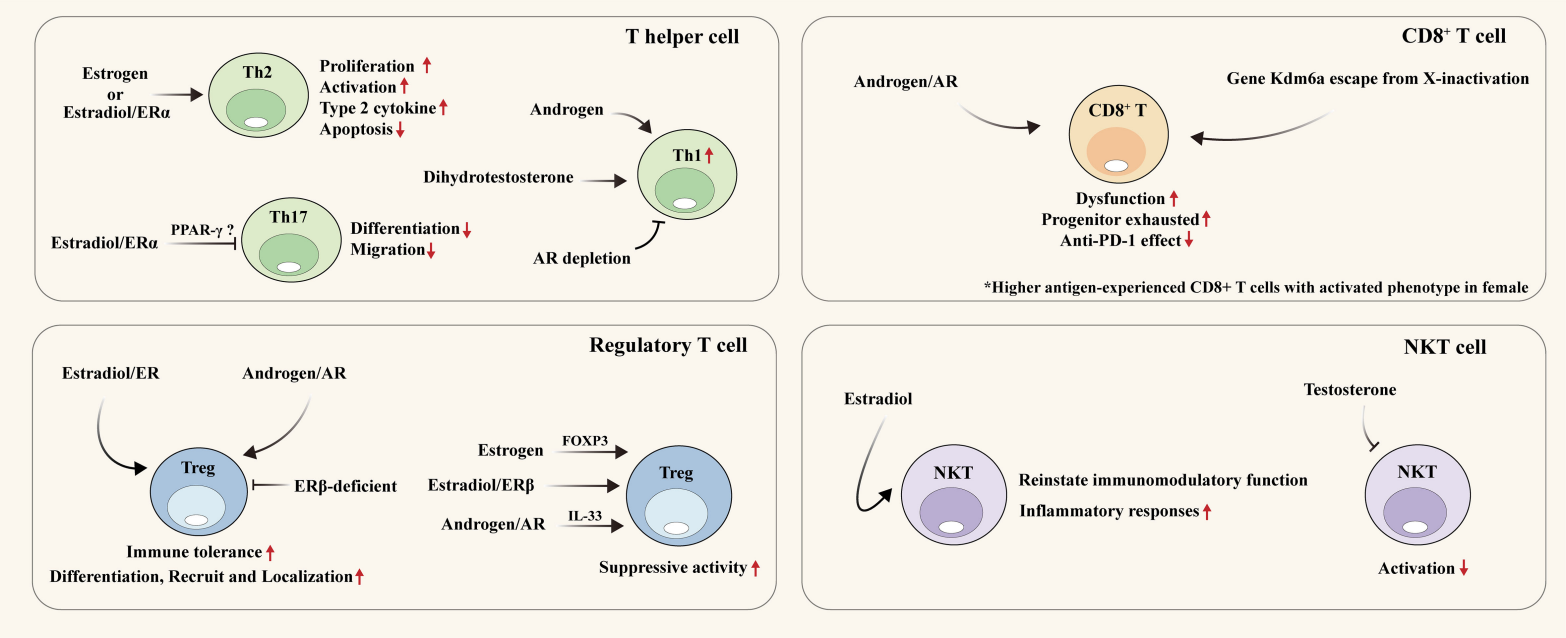
Sexual dimorphism of T cell. Sex hormones on various T-cell types, including T helper cells, CD8+ T cells, regulatory T cells, and NKT cells. Th, T helper cell; ER, Estrogen receptor; AR, Androgen receptor; PPAR-γ, Peroxisome proliferator-activated receptor gamma; PD-1/PD-L1, Programmed death receptor 1/programmed cell death 1 ligand 1; Treg, Regulatory T cell; FOXP3, Forkhead box P3; IL, Interleukin; NKT, Natural killer T cell.

T cells, the critical component of adaptive immune system, play an important role in immune surveillance and pathogen elimination through their diverse subtypes, including CD4^+^ T cells (T helper cells (Th) and Tregs and CD8^+^ cytotoxic T cells. Studies have shed light on potential sex-based disparities in T cells, which participate in the development and progression of various diseases (16, 85, 86). Mechanistically, T cell’s state and function can be largely regulated by estrogen or androgen signaling (87, 88), and even X/Y chromosome factors, affecting immune responses in physiological and pathological conditions (89, 90). Therefore, understanding the sex divergence of T cells is paramount for developing effective therapies for distinct diseases (**Figure 3**) (**Table 3**).

**Table 3:** Sex difference of T cells.

Cell	Etiology	Model/Source	Effects	References
Th	Asthma	Human peripheral T cell	Estrogen increases type 2 cytokine production and reduces Th2 cell apoptosis	Vijeyakumaran et al., 2023 (16)
	Bone loss	Mouse	Estrogen deficiency enhances antigen presentation and T cell activation	Cenci et al., 2003 (92)
	Cerebral aneurysm rupture	Mouse	Estrogen deficiency intensifies circulating Th17 cells and IL-17A levels	Hoh et al., 2018 (93)
	Autoimmune disease	CD4-*PPARγ*^KO^ mice	PPARγ restrains Th17 differentiation in male while regulates Th1, Th2, and Th17 differentiation in female	Park et al., 2016 (94)
	Colitis	CD4-creERα^fl/fl^ mice, T cell-deficient mouse	ERα deletion reduces T cell proliferation and activation	Mohammad et al., 2018 (95)
	Collagen-induce arthritis	Mouse	E2-ERα mitigates the migration of Th17 cells	Andersson et al., 2015 (96)
	Autoimmune encephalomyelitis	Mouse	E2-ERα limits Th17 cell differentiation	Garnier et al., 2018 (97)
	Asthma	Mouse	Dihydrotestosterone decreases while AR depletion aggravates Th2 inflammation	Ejima et al., 2022 (98)
	Physiology	Human peripheral T cell	Androgen increases Th1 responses in male	Girón-González et al., 2000 (100)
Treg	Autoimmune encephalomyelitis	Mouse	E2 promotes immune tolerance via expanding the Treg compartment	Polanczyk et al., 2004 (101)
	Obesity	Mouse	E2 modulates Treg localization in adipose tissue	Ishikawa et al., 2020 (102)
	Autoimmune encephalomyelitis	Mouse	Estrogen elevates FOXP3 level and immunosuppressive activity of Treg	Polanczyk et al., 2005 (103)
	Ileitis	*Erα*^-/-^, *Erβ*^-/-^ mouse	ERβ knockout impairs Treg differentiation	Goodman et al., 2020 (104)
	Pneumococcal pneumonia	Mouse	E2-ERβ axis augments Tregs number with intensified immunosuppressive effect	Xiong et al., 2021 (105)
	Allergic airway inflammation	Mouse	AR enhances the stability and suppressive function of Tregs and protects against type 2 inflammation	Gandhi et al., 2022 (106)
	Adipose tissue inflammation	Mouse	Androgen recruits and locally expands Treg cell number in adipose tissue	Vasanthakumar et al., 2020 (107)
CD8^+^ T	Bladder carcinogenesis	Mouse	Androgen-AR promotes dysfunction and exhaustion of CD8^+^ T cell	Kwon et al., 2022 (109)
	Prostate cancer	Human, mouse	Blocking AR restores CD8^+^ T cell function in male	Guan et al., 2022 (110); Yang et al., 2022 (111)
	Non-small cell lung cancer	Human, mouse	Androgen-AR attenuates NF-ĸB-associated anti-tumor response in T cells in male	Zhang et al., 2023 (112)
	Glioblastoma	Mouse	More functionally exhausted CD8^+^ T cells in male due to *Kdm6a* escape from the X-inactivation	Lee et al., 2023 (113)
	*Coxsackievirus B3* infection	Mouse	Increased activated CD8^+^ T cells in female	Dhalech et al., 2023 (114)
NKT	Immunological hyperactivity	Mouse	Estrogen regulates IL-4 and IFNγ production in iNKT cells and participates in Th1/Th2 differentiation	Gourdy et al., 2005 (118)
	Non-obese diabetes	*Jα18*^−/−^ mouse	E2 reinstates the immunomodulatory function of iNKT cell	Gourdy et al., 2016 (119)
	Steatohepatitis	Mouse	NKT cell depletion exacerbates leukocyte infiltration and inflammatory liver injury in male	Cuño-Gómiz et al., 2023 (120)
	Amebic liver abscess	Mouse	Testosterone inhibits NKT cell activation	Lotter et al., 2013 (121)
	Influenza A virus infection	Mouse	Expansion of iNKT cell population with boosted inflammation in female	Humeniuk et al., 2023 (122)

Th, T helper cell; IFN, Interferon; IL, Interleukin; E-cadherin, Epithelial cadherin; PPAR-γ, Peroxisome proliferator-activated receptor gamma; ER, Estrogen Receptor; Treg, Regulatory T cell; FOXP3, Forkhead box P3; AR, Androgen Receptor; NKT, Natural Killer T cell; E2, Estradiol.

#### 3.3.1 Th cell

Th cells are a pivotal subset of CD4^+^ T cells that orchestrate immune responses by providing essential signals to other immune cells. These cells differentiate into various subtypes, divided into Th1, Th2, Th17 and etc., each defined by distinct cytokine profiles and function (91). Among various sex factors that affect Th cells, estrogen has been extensively studied and was found to increase type 2 cytokine production and reduce Th2 cell apoptosis, leading to the airway inflammation in severe asthma (16). Otherwise, estrogen deficiency is found to upregulate IFN-γ-induced class II trans-activator, enhance antigen presentation and T cell activation, resulting in bone loss (92). Estrogen shortage also increases the risk of cerebral aneurysm rupture by intensifying levels of circulating Th17 cells and IL-17A levels, as well as downregulating E-cadherin expression (93). *Peroxisome proliferator-activated receptor gamma* has recently been highlighted a key modulator of sexual differences in adaptive immunity and autoimmune diseases. It only restrains Th17 differentiation in male T cells while regulates the differentiation of Th1, Th2, and Th17 in female T cells according to distinct estrogen levels (94). To further investigate the underlying mechanism, researcher have constructed T cell-specific *ERα* knockout mice and found that ERα deletion reduced T cell proliferation and activation, contributing to their decreased pathogenicity in a murine model of colitis (95). In collagen-induced experimental arthritis, estradiol treatment potently mitigates its severity via regulating the migration of Th17 cells in an ERα-dependent manner (96). Meanwhile, estradiol-ERα axis in FOXP3^neg^ CD4^+^ T cells limit Th17 cell differentiation, thus inhibiting the development of autoimmune encephalomyelitis in the mouse model (97). Furthermore, androgen signaling also plays a significant role in the sex-based regulation of Th cells. Scholars have discovered that dihydrotestosterone administration could decrease while AR depletion in T cells aggravated Th2 inflammation by inducing the expression of dual specificity phosphatase-2 in a murine model of acute allergic asthma (98). Other studies also pointed that androgen can increase Th1 responses in males (99, 100). The evidence may also explain the sex bias of asthma post adolescence. Notably, estrogen’s effect on Th17 differentiation differs by disease context: it restrains Th17 in autoimmune encephalomyelitis but promotes Th17 in cerebral aneurysm rupture, likely due to disease type and local estrogen concentration, reflecting the context-dependent nature of sex hormone-immune crosstalk.

#### 3.3.2 Tregs

Tregs are a specialized subset of CD4^+^ T cells and play a critical role in maintaining immune homeostasis and preventing autoimmunity. Exogenous estradiol has been shown to promote immune tolerance via expanding the Treg compartment (101). It can also modulate chemokine signals associated with Treg localization and mitigate obesity-related chronic inflammation (102). Additionally, CD4^+^CD25^+^ T cells from pregnant and estrogen-treated mice exhibit elevated FOXP3 level and enhanced suppressive activity, controlling abnormal inflammatory responses (103). Compared with males, female patients with Crohn’s disease exhibit decreased ERβ expression on T cells in both the ileal mucosa and peripheral blood. ERβ-deficient mice display impaired Treg differentiation followed by a female-specific exacerbation of intestinal inflammation (104). In male mice with severe pneumococcal pneumonia, estradiol-ERβ axis alleviates lung inflammation and tissue injury by augmenting the number of Tregs with their intensified immunosuppressive effect (105). Studies have also highlighted the effects of AR signaling on Tregs. It is capable of enhancing the stability and suppressive function of Tregs via IL-33/suppression of tumorigenicity 2 (ST2) pathway, thereby safeguarding against the progression of allergen-induced type 2 inflammation (106). Recently, sexual dimorphism of Tregs in the visceral adipose tissue has been uncovered and is determined in a sex hormone-dependent manner. Androgen actively recruits and locally expands Treg cell numbers, which regulates immune responses and limits adipose tissue inflammation (107).

#### 3.3.3 CD8^+^ T cell

CD8^+^ T cells, also known as cytotoxic T lymphocytes, are another crucial subtype of T cells primarily responsible for identifying and eliminating cancerous or infected cells (108). So far, numerous studies have focused on sexual disparities in development and progression of certain nonreproductive system cancers, with the underlying mechanisms remain enigmatic. In preclinical cancer models, androgen-AR axis is proved to promote CD8^+^ T cell dysfunction and contribute to the male bias in the frequency of progenitor exhausted CD8^+^ T cells in the tumor microenvironment (109). Other studies also concentrate on the regulation of androgen signaling on T cell exhaustion and indicate that blocking AR could restore CD8^+^ T cell function and enhance responsiveness to anti-programmed death receptor 1 (PD-1)/PD-L1 therapy in males (110, 111). Likewise, activation of androgen-AR signaling pathway attenuates nuclear factor kappa-B-associated anti-tumor response in T cells and anti-PD-1 effects in male mice, which can be reversed through either pharmacological intervention or surgical castration (112). Another noteworthy study has revealed a worse prognosis in male glioblastoma patients than females, attributing to more tendency of functionally exhausted CD8^+^ T cells in the male. Mechanistically, the gene *Kdm6a* that escape from X-inactivation, rather than sex hormone, plays a role in the sexual dimorphism of T cell-mediated anti-tumor immunity (113). In addition, sex bias in CD8^+^ T cells is also observed in other diseases. *Coxsackievirus B3* triggers the expansion of antigen-experienced CD8^+^ T cells with an activated phenotype in female mice, whereas male mice exhibit no significant changes in T cell-related immune responses (114). Beyond viral infections, sex differences in CD8^+^ T cells also contribute to chronic pain, a condition predominantly affecting women: Studies indicate that in females, Pannexin-1 channel-expressing microglia and T cells differentially induce mechanical hyperalgesia, with female-derived CD8^+^ T cells showing a greater tendency to release leptin, as pro-inflammatory adipokine, further enhances pain sensitivity (115). This heightened immunity in females facilitates more effective pathogen elimination but also a predisposition to autoimmune diseases.

#### 3.3.4 Natural killer T cell

Natural killer T cell (NKT) represents a special subpopulation of T cells with both T cell receptors and NK cell receptors on the surface (116, 117). These cells can rapidly release numerous cytokines and play a bridging role between the innate and adaptive immunity. Currently, attentions have been paid on sex differences of NKT cells in several physiological and pathological conditions. Estrogens have the ability to regulate the production of IL-4 and IFNγ in invariant NKT (iNKT) cells, which participate in the regulation of Th1/Th2 differentiation (118). The subsequent research has revealed that estradiol could reinstate the immunomodulatory function of iNKT cells in non-obese diabetic mice, and might be beneficial to insulin secretion in type 1 diabetes (119). Other study demonstrates that male steatohepatitis mice lacking NKT cells experience exacerbated infiltration of neutrophil and macrophage, resulting in more severe inflammatory liver injury compared to females with the known protection provided by estrogens (120). Amebic liver abscess has shown strong preferences for adult males. By use of the mouse model, researcher have discovered that testosterone inhibited the activation of NKT cells, manifested by IFN-γ generation, thereby promoting development of the abscesses (121). Contrarily, influenza A virus infection leads to higher mortality in women of reproductive age. In the infectious murine model, female mice exhibit a sex-dependent expansion of iNKT cell populations in the lung and liver, along with boosted inflammatory responses compared with males, although the underlying mechanism remains unclear (122).

Together, estrogen governs Th cell polarization and Treg expansion via ERα/PPAR-γ signaling, androgen promotes CD8^+^ T cell exhaustion through AR/PD-L1 axis, and both hormones regulate NKT cell cytokine secretion (IL-4/IFN-γ), collectively mediating sex dimorphism in T cell subsets. These mechanisms contribute to female-biased autoimmune diseases (e.g., SLE, rheumatoid arthritis) driven by Th17 overactivation, reduced anti-tumor immunotherapy efficacy in males (due to CD8+ T cell exhaustion), and sex differences in viral infection outcomes (e.g., Coxsackievirus B3) and chronic pain.

### 3.4 NK cell

**Figure 4:**
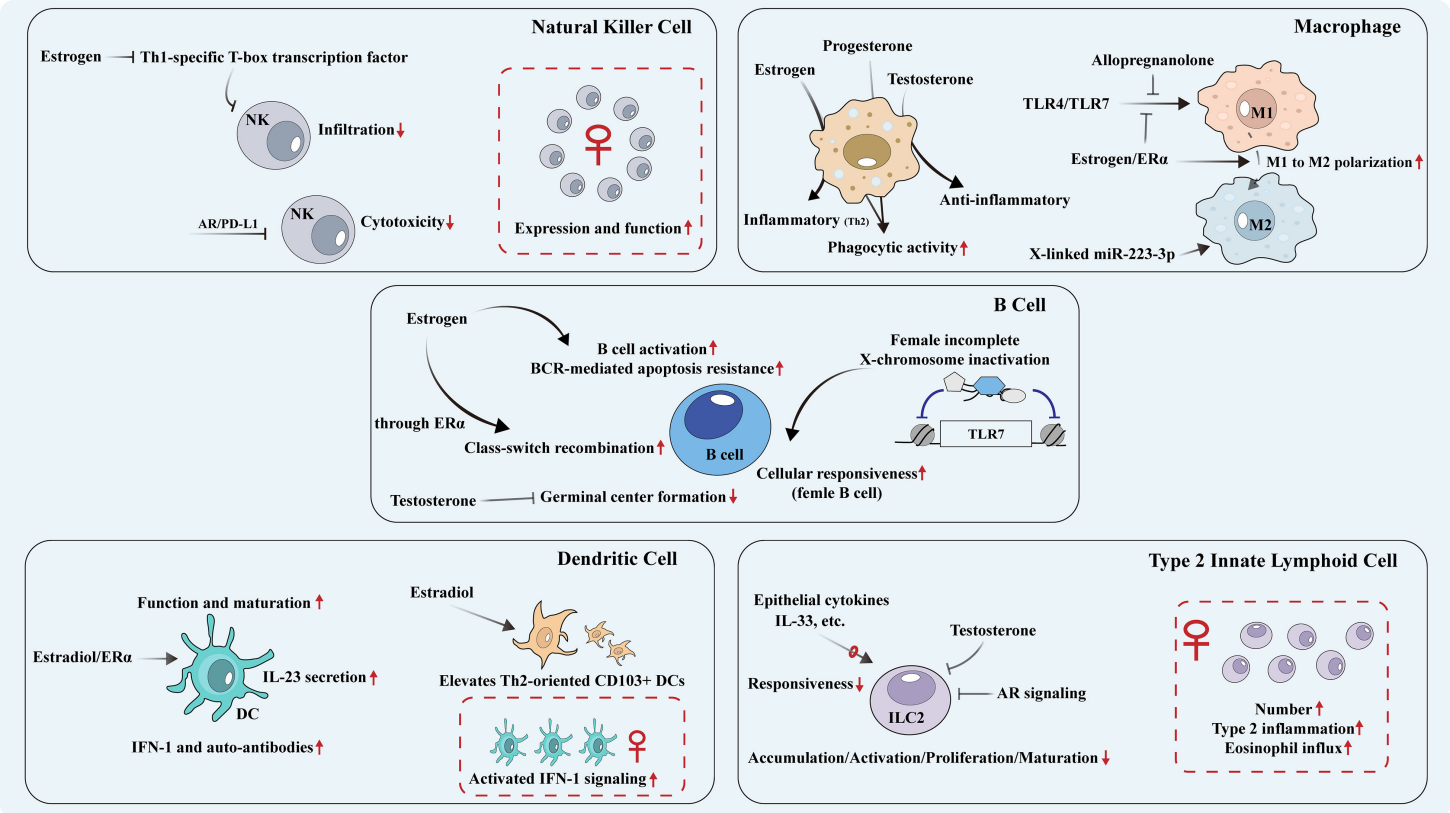
Sexual dimorphism of other immune cells. The effects of sex hormones on various immune cells along with the differences in immune responses associated with biological sex. Th, T helper cell;ER, Estrogen receptor; AR, Androgen receptor;NK, Natural killer cell; PD-1/PD-L1, Programmed death receptor 1/programmed cell death 1 ligand 1; M, Macrophage; IFN, Type I interferon; TLR, Toll-like receptor; IL, Interleukin; DC, Dendritic cell; ILC2, Group 2 innate lymphoid cell; HMGB1, high mobility group box 1; HIF, hypoxia-inducible factor; BCR, B cell receptor; TLR, Toll-like receptor.

NK cells are innate lymphocytes that primarily exhibit cytotoxic effects by producing perforin, granzyme and IFN-γ in response to pathogens and malignant cells (123). However, research on their sexual dimorphism remains limited. Based on the observation that Coxsackievirus B3-associated myocarditis predominantly affects males, Zhou et al. explore that estrogen inhibits the expression of Th1-specific T-box transcription factor and the infiltration of cardiac *IFN-γ^+^* NK cells, thereby preventing myocarditis in female (124). In Parkinson’s mice, NK cell from young female presents a higher expression of its receptors and IFN-γ production, which becomes less pronounced with aging (125). Androgens can also influence the function of NK cells. Elevated level of dihydrotestosterone reduces the cytotoxicity of NK cells, leading to their diminished ability to eliminate castration-resistant prostate cancer cells through the AR/PD-L1 signaling pathway (126). Furthermore, Liu et al. demonstrate that targeting AR, via either antiandrogen therapy or AR depletion, could enhance the tumor-killing efficacy of NK cells due to the decreased PD-L1 expression in bladder cancer (127) (**Figure 4**) (**Table 2**). Therefore, in NK cell, estrogen inhibits IFN-γ+ NK cell infiltration via suppressing Th1-specific transcription factors, while androgens reduce NK cell cytotoxicity through AR/PD-L1 signaling, leading to sex-specific NK cell functional differences. These differences explain the male predominance in Coxsackievirus B3-induced myocarditis and castration-resistant prostate cancer, as well as the potential of anti-androgen therapy to enhance NK cell-mediated tumor killing in bladder cancer.

### 3.5 Macrophage

Macrophages are versatile immune cells with diverse functions, mainly including eliminating pathogens, presenting antigens, and producing cytokines. They can polarize into pro-inflammatory (M1) or anti-inflammatory (M2) state, adapting to environmental signals to regulate immune response, tissue repair, and homeostasis (128). The regulation of sex-determining factors on macrophage function is diverse and complex. In infectious diseases, Q fever influences more males than females, despite a similar level of Coxiella burnetii exposure. It has been reported that the protective role of estrogen is achieved by enhancing the inflammatory profile of macrophages, whereas testosterone might affect disease progression via fostering an anti-inflammatory response (129). As one of the most important antibacterial mechanisms, phagocytic activity is markedly diminished in peritoneal macrophages from female mice subjected to ovariectomy compared with the sham group (57), while can be enhanced through treatment with estradiol or progesterone (130).

However, in inflammatory reactions, the situation is completely different. Estrogen-ERα axis exerts inhibitory action on the activation of TLR4 signaling and macrophage-associated inflammation, thereby promoting the stability of atherosclerotic plaques post-menopause (131). Another study claims that estradiol negatively modulates TLR4-induced M1 polarization and increases Th2 immune responses by activating serum-glucocorticoid regulated kinase 1 at the maternal-fetal interface, thus avoiding recurrent pregnancy loss (132). Besides, the anti-inflammatory role of allopregnanolone has also been identified in human macrophages via the inhibitory effects on both TLR4 and TLR7 activation, which lead to cytokine/chemokine reduction, particularly in female donors (133). When fed with high fat diet, ovariectomy increases the risk of non-alcoholic fatty liver disease in female mice. This is probably due to the inhibited recruitment and activation of M1 macrophages by estrogen-ERα signaling via the downregulation of CCR2 (134). In a mouse model of postmenopausal chronic stress, estradiol supplementation promotes the polarization of M1 to M2 macrophages in a β2-adrenoceptor-dependent manner, therefore facilitating the resolution of myocardial inflammation (135). Asthma is a chronic Th2 inflammation of the lung that affects more women than men in adulthood, with alveolar macrophage emerging as predominant mediator of the allergic inflammation. Estrogen-ERα axis is demonstrated to enhance IL-4-induced M2 polarization in asthmatic mice of both sexes challenged by ovalbumin (136, 137). Other findings also highlight the stronger IL-4 responsiveness in macrophages from female asthma patients than the males, leading to increased monocyte recruitment and M2 polarization in this disease (138). Sexually dimorphism is also prominent in pulmonary fibrosis, 5-hydroxytryptamine receptor 2C^+^alveolar macrophages specifically secrete angiopoietin-like protein 4, which mediates macrophage metabolic reprogramming, promoting lipolysis and inhibiting glycolysis, thereby suppressing grancalcin expression, reducing its accumulation in the lungs of female mice, and alleviating the pathological progression of pulmonary fibrosis (139). Notably, external stimuli like chemotherapy also trigger sex-specific macrophage responses: female macrophages upregulate intercellular adhesion molecule-1 more rapidly upon exposure, enhancing pro-inflammatory M1 polarization and release of pro-nociceptive factors and amplifying macrophage-peripheral nociceptor crosstalk, accelerating nociceptor activation and earlier onset of chemotherapy-induced neuropathic pain hallmarks (e.g., mechanical allodynia) in females (140). Further studies are needed to determine how macrophage polarization is specifically instructed by the pathological state (infection vs. autoimmunity) and local cytokine signals.

It is noteworthy that sex bias in macrophages not only attributes to sex hormones but may also be due to the chromosomes. Deny et al. discover that female macrophages exhibit higher level of chromosome X-linked *microRNA-223-3p* (*miR-223-3p)*, which negatively correlates with pro-inflammatory M1 phenotype in *Streptococcus agalactiae*-associated pneumonia (141) (**Figure 4**) (**Table 2**).

Collectively, estrogen regulates macrophage polarization (M1/M2) via ERα/TLR4 and serum-glucocorticoid regulated kinase 1 pathways, testosterone fosters anti-inflammatory macrophage responses, and X-chromosome-linked miR-223-3p in females negatively correlates with M1 phenotype, jointly mediating sex bias in macrophage function. These regulatory effects underlie sex differences in Q fever susceptibility (male predominance), postmenopausal atherosclerotic plaque stability, asthma severity (female predominance), and non-alcoholic fatty liver disease risk.

### 3.6 Dendritic cell

Dendritic cells (DCs) are antigen-presenting cells and pivotal for initiating immune responses and maintaining immune tolerance (142). The action of sex-dependent DC functions in health and disease is complicated. Research has shown that ERα S-glutathionylation mediated by glutathione S-transferase Pi controls the differentiation and metabolic function of mouse dendritic cells (143). The estradiol-ERα signaling contributes to the strong sex bias on the capacity of plasmacytoid DC to produce IFN-1 in response to TLR7 (144, 145). Thus, targeting the ER signaling pathway in plasmacytoid DCs exhibits inhibitory effects on pathogenic generation of IFN-1 and autoantibodies, thereby preventing the progression of lupus-like syndrome in the initial stage of SLE (146, 147). The sexual dimorphism of DC function also appears in psoriasis, autoimmune encephalomyelitis and asthma. Selective ERα agonist enhances IL-23 secretion in DCs under LPS stimulation and aggravates pruritic responses in a psoriasis mouse model initiated by imiquimod (148). In encephalomyelitis, ERα signaling suppresses the neurotoxic function and maturation status of DC, which in turn impacts IL-12 and IFN-β production and ultimately modulates disease progression (149). With regard to asthma, estradiol elevates Th2-oriented CD103^+^ DCs in the bronchial lymph node, which may explain the higher prevalence and severity in female patients compared with the males (150). However, the activated IFN-1 signaling in bone marrow-derived DCs from 7-day-old female mice can also be protective in respiratory syncytial virus-related pneumonia, suggesting the diversity of sex dimorphism in DCs (151) (**Figure 4**) (**Table 4**). Jointly, estradiol-ERα signaling modulates plasmacytoid DC IFN-1 production and CD103^+^ DC Th2 polarization, while ERα S-glutathionylation controls DC differentiation and metabolic function, contributing to sex-specific DC-mediated immune responses. This sex dimorphism explains the female predominance in SLE (linked to pathogenic IFN-1 production by plasmacytoid DCs), psoriasis, and adult asthma, as well as the protective role of DC IFN-1 signaling in female respiratory syncytial virus-related pneumonia.

**Table 4:** Sex difference of DC, ILC2 and B cell.

Cell	Etiology	Model/Source	Effects	References
DC	*Lipopolysaccharide* infection	Mouse	ERα S-glutathionylation controls DC differentiation and metabolism	Zhang et al., 2018 (143)
	Immune-related disease	Human, mouse	E2-ERα signaling contributes to the strong sex bias on the pathogenicity of plasmacytoid DC	Meier et al., 2009 (145); Seillet et al., 2012 (144)
	SLE	Mouse	ER signaling elevates pathogenic generation of IFN-1 and auto-antibodies in plasmacytoid DC	Rowland et al., 2014 (147); Sisirak et al., 2014 (146)
	Psoriasis	Mouse	ERα agonist enhances IL-23 secretion in DC under LPS stimulation	Iwano et al., 2020 (148)
	Encephalomyelitis	Mouse	ERα signaling suppresses neurotoxic DCs’ function and maturation	Khaw et al., 2023 (149)
	Asthma	Mouse	E2 elevates Th2-oriented CD103^+^ DCs in the bronchial lymph node in female	Masuda et al., 2018 (150)
	RSV-related pneumonia	Mouse	Activated IFN-1 signaling in bone marrow-derived DC is protective in female	Malinczak et al., 2023 (151)
ILC2	Asthma	Human	Elevated level of circulating and sputum ILC2s in female	Aw et al., 2019 (154); Cephus et al., 2017 (153)
	Asthma	Human, mouse	Higher levels of ILC2s and Th2 cytokines in female mice; testosterone reduces the proliferation of ILC2s and their production of type 2 cytokines	Wang et al., 2020 (155)
	Asthma	Mouse	Heightened type 2 inflammation with increased eosinophil influx and expansion of ILC2 in female	Zhao et al., 2019 (156)
	Asthma	Mouse	AR signaling limits the expansion, maturation and function of ILC2	Laffont et al., 2017 (157)
	Skin immunity	ILC2-deficient transgenic mouse	Androgen reduces ILC2 and the accumulation and activation of DC	Chi et al., 2024 (158)
B cell	Immune-related disease	Mouse	E2 reduce B cell apoptosis/activation	Grimaldi et al., 2002 (162)
	Immune-related disease	Mouse	E2-ERα signaling increase capacity for class-switch recombination	Hill et al., 2011 (166)
	Physiology	Mouse	testosterone tend to exert immunosuppressive effects on B cells	Zhao R, et al. 2020 (167); Wilhelmson et al. 2018 (168)
	SLE	Human peripheral B cell	Escape of X-inactivation leads to dual-allelic expression of TLR7 in B cells and monocytes, resulting in cell-intrinsic hyperresponsiveness	Souyris et al., 2018 (169)
	Physiology	GM and K562 cell lines	Loss of XIST-mediated silencing in B cells leads to expansion of atypical B cells	Yu et al., 2021 (170)

E2, Estradiol; AR, Androgen Receptor; ER, Estrogen Receptor; IFN, Interferon; IL, Interleukin; ILC2, Type 2 Innate Lymphoid Cell; DC, Dendritic Cell; SLE, Systemic Lupus Erythematosus; RSV, Respiratory Syncytial Virus; TLR, Toll-like receptor; XIST, X-inactive specific transcript.

### 3.7 Group 2 innate lymphoid cell

Group 2 innate lymphoid cells (ILC2s) are a unique subpopulation of immune cells with lymphocyte morphology but do not express lineage markers. ILC2s possess strong immune regulatory functions on allergic inflammation and tissue repair (152). Sexual dimorphism can be observed in ILC2s, though the conclusion is controversial. These conflicting ILC2 findings (elevated vs. no difference in asthma) may stem from asthma severity, sample source (circulating vs. sputum), or hormonal status (reproductive vs. post-menopause). Cephus et al. (153) claimed an elevated level of circulating ILC2s in women with moderate to severe asthma, while Aw et al. (154) observed higher sputum ILC2 numbers in female mild asthmatics. Yet, Wang et al. (155) reported no significant sex-based difference in the numbers of ILC2s in patients with asthma or allergic rhinitis (155). In asthmatic mouse model initiated by ovalbumin+IL-33, the females exhibit heightened type 2 inflammation, along with increased eosinophil influx and expansion of ILC2s, which may be attributed to greater IL-13 production compared to the males (156). Consistent with this finding, Wang et al. affirm higher levels of ILC2s and Th2 cytokines in female mice with the induction of IL-33 compared to the males. They further find a crucial protective role of testosterone in this allergic disease model (155). Mechanistically, AR signaling hinders the expansion and maturation of ILC2 and limits their responsiveness to IL-33 in type 2 airway inflammation (157). A more recent study published in Science try to unveil the mechanism underlying the sexual immune dimorphism: androgens negatively regulate ILC2 and the accumulation and activation of DCs in the skin, leading to the reduced tissue immunity in males (158) (**Figure 4**) (**Table 4**). In conjunction, androgen-AR signaling restrains ILC2 expansion, maturation, and responsiveness to IL-33, while female mice exhibit heightened ILC2-mediated type 2 inflammation, leading to sex differences in ILC2 function. These differences contribute to the higher prevalence and severity of asthma in females, as well as reduced tissue immunity in males (mediated by androgen-dependent ILC2 suppression).

### 3.8 B cell

B cells are central to humoral immunity, primarily functioning through antigen presentation, cytokine secretion, and antibody production. Significant sexual dimorphism exists in B cell biology, with females generally mounting stronger humoral responses than males. This is evidenced by higher baseline levels of immunoglobulins and more robust antibody production after vaccination or infection (159, 160). The enhanced B cell activity in females is a double-edged sword, contributing to both superior antiviral protection and a markedly higher susceptibility to systemic autoimmune diseases like SLE, where pathogenic autoantibodies are central (161).

The mechanisms are multifaceted, involving both hormonal and genetic pathways. At the hormonal level, estrogen promotes B cell activation and antibody diversification. It alters thresholds for B cell apoptosis and activation and has been shown to protect purified B cells from B cell receptor-mediated apoptosis (162). Concurrently, *in vitro* studies demonstrate that estrogen can significantly enhance antibody production, increasing the output of immunoglobulin G (IgG) and IgM from human B cells by more than two-fold (163). The hormone drives antibody diversification primarily through ER, which binds directly to the immunoglobulin heavy chain locus to participate in the three-dimensional DNA looping required for class-switch recombination, thereby influencing antibody isotype selection. This genomic action is specific to ERα, which is uniquely capable of disrupting B cell tolerance and permitting the survival of high-affinity autoreactive clones, directly linking this mechanism to female-biased autoimmunity (164–166). In contrast, testosterone tends to exert immunosuppressive effects, such as inhibiting germinal center formation (167, 168).

At the genetic level, contributions to the female bias in autoimmunity include incomplete X-chromosome inactivation, leading to biallelic expression of immune-related genes like *Tlr7* in a subset of female immune cells. This results in a higher gene dosage and enhanced cellular responsiveness, such as lowered activation thresholds in B cells (169). Crucially, the maintenance of X-inactivation itself is actively regulated in B cells. Recent work shows that a B cell-specific *X-inactive* sp*ecific transcript* complex enforces this silencing, and its dysfunction results in the expansion of pathogenic atypical B cells, directly linking the stability of X-inactivation to autoimmune B cell responses (170). These genetic mechanisms provide an explanation for the female bias in autoimmunity that is independent of, but potentially synergistic with, sex hormone effects. Additionally, B cells exhibit sex-based disparities in tumor immunity, and the regulation of B cell malignancy by sex hormones has been comprehensively reviewed elsewhere (159).

## 4 Modulatory effects of regulatory variables on sexual dimorphism of immune cells

### 4.1 Age, menopause and andropause

Previous study pointed that women consistently generate at least twice the amount of influenza vaccine-induced antibodies as men. Intriguingly, age acts as a key modifier of this sexual dimorphism. Immune and inflammatory responses are more robust in boys prior to puberty, with this advantage shifting to adult women post-puberty (171). Age-related hormonal shifts occur in both sexes: females experience reduced estrogen and progesterone, while males see declining testosterone. Such age-related hormonal decline during menopause and andropause directly reshapes immune cell functions by modulating sex hormone signaling (171–174). In detail, for menopause (female), postmenopausal estrogen drops by 60%-80%, reducing bone marrow GM-CSF secretion, impairing neutrophil maturation (25% lower mature proportion) and bacterial phagocytosis (52). Additionally, B and T cell counts decline (with fewer circulating lymphocytes vs. younger women), pro-inflammatory cytokines (IL-1β, IL-6, tumor necrosis factor-α) are markedly elevated, and T cell apoptosis increases following natural or surgical menopause. Estrogen-containing hormone replacement therapy in postmenopausal women improves immune function by increasing circulating B cell counts and reducing baseline pro-inflammatory cytokine levels, relative to non- hormone replacement therapy users. For andropause (male), studies indicate that low testosterone impairs CD4^+^T cell counts, Th1 responses, and Treg function; however, the impact of testosterone replacement therapy on immunity in aged men remains unreported (175–177).

### 4.2 Environmental exposures

Environmental factors serve as another critical modulator that can amplify or alter inherent immune sexual dimorphisms by interfering with sex hormone signaling pathways. The core mechanism involves environmental pollutants acting as endocrine disruptors, which can competitively bind to sex hormone receptors or affect their expression, thereby dysregulating normal hormone-immune communication (178). For instance, cigarette smoke may suppress the expression of ERα in lung tissue, attenuating the estrogen-mediated enhancement of neutrophil phagocytic function and consequently reducing the antibacterial capacity in female smokers. This is supported by clinical research showing that smoking causes a statistically significant decrease in phagocytic activity specifically in female subjects, impairing host defense functions (179, 180). Similarly, pollutants like PM2.5 are implicated in potentiating the inhibitory effects of androgens on immune cells such as NK cells, which may contribute to a gender-differential susceptibility to diseases like lung cancer (179). Animal studies reveal that the inflammatory response to particulate matter differs fundamentally by sex: exposed male mice show markers linked to acute inflammation (e.g., IL-1β, cyclooxygenase-2), whereas females exhibit increases in mediators like IL-17 that may promote chronic inflammation and tissue remodeling. Further evidence demonstrates that exposure to environmental stressors—including chlorine inhalation injury or prenatal stress, can lead to significant sex-based differences in immune cell migration, activation, and inflammatory responses (59, 178, 181).

### 4.3 Lifestyle interventions

Lifestyle factors are increasingly recognized as potential modulators of sexual dimorphism in immune responses, with growing evidence revealing sex-specific nuances in immune cell responses to these interventions beyond shared regulatory goals. For instance, dietary patterns such as high-fat diets can activate pathways that preferentially enhance estrogen-mediated M2 polarization of macrophages in females, contributing to differential metabolic disease susceptibility (182, 183). Similarly, the immunomodulatory effects of physical activity exhibit distinct sexual dimorphism (184). In males, aerobic exercise can elevate testosterone, which acts directly on CD4^+^T lymphocytes to increase the production of the anti-inflammatory cytokine IL-10 (185). Concurrently, exercise boosts Tregs’ frequency and suppressive capacity with post-pubertal males typically having more abundant, suppressive circulating Tregs than females (186, 187). Meanwhile, males show greater immune sensitivity to stress, due to androgens activating corticotropin-releasing hormone neurons expressing androgen receptors, enhancing hypothalamic-pituitary-adrenal axis activity and glucocorticoid release, which in turn triggers more intense apoptosis of immune cells like thymic T cells (188). These findings underscore that the impact of common lifestyle factors, though broadly beneficial, is precisely fine-tuned by biological sex at the level of specific immune cell subsets and functions.

## 5 Concluding remarks

For decades, sex disparities exist and affect health and disease, yet underlying mechanisms are largely understudied, including the synergistic regulatory effects between sex hormones and X/Y chromosome genes which remain to be deciphered. Our review undertakes a comprehensive synthesis, focusing on sex as a pivotal regulator in both physiological processes and pathological states. We expound upon various factors, including hormones and chromosomes, and dissect their profound impacts on physiology and diseases through immune cells. The aim is to elucidate sex - specific responses, thereby paving the way for the implementation of sex - tailored precision medicine. The aforementioned research also provides clinically transferable insights, as using sex hormones to modulate neutrophil functions and apoptosis may impede disease progression; or combining sex hormones with immune checkpoint inhibitors may enhance the anti-tumor abilities of cells, such as CD8^+^T and Tregs (as a typical example of translating hormone-immune axis mechanisms into clinical applications). Further exploration of sex dimorphism in emerging immune cell subsets, such as ILC1/ILC3 and γδT cells identified in recent years, will fill critical gaps in understanding sex-biased immunity and disease.

Although research efforts have increasingly illuminated the underlying mechanisms, further advancements in depth and methodology are essential. We anticipate novel studies should consider four key directions: Firstly, as current understanding of sex differences is shallow, with the rapid progress of life sciences and biotechnology, new techniques like single-cell RNA sequencing and clustered regularly interspaced short palindromic repeats-Cas9 gene editing can seek a breakthrough. The former helps explore cell characteristics related to sex differences, and the latter enables precise gene modifications in sex-related processes. Secondly, targeting hormone-immune axes for precision medicine. Regarding the application of sex hormones or agonists and inhibitors of their signaling pathways, the main problem is the overabundance of drug targets, resulting in significant side effects. Hence, it is crucial to develop targeted approaches like nano-delivery systems, which can proficiently modulate sex-specific cellular responses and minimize the incidence of adverse side effects. Thirdly, clinical studies should integrate hormonal fluctuations across key life stages (125) (as adolescence, reproductive, menopause) and consider ‘gender’ identity when assessing treatment responses. This will help tailor interventions to the specific needs of diverse patient populations, ensuring more precise and effective care. Lastly, policy and clinical practice must address the unique health needs of women, particularly in light of sex-specific disease outcomes. Efforts should also focus on raising awareness about sex differences in medical care, using research findings to guide policy decisions and reduce health disparities. By pursuing these directions, we can enhance our understanding of sex differences in cells, health and disease, ultimately leading to more personalized and equitable healthcare strategies.
